# Sensitivity analysis of operation parameters of the salt cavern under long-term gas injection-production

**DOI:** 10.1038/s41598-023-47352-w

**Published:** 2023-11-16

**Authors:** Huabin Zhang, Peng Wang, Qiqi Wanyan, Kang Li, Kai Gao, Xianru Yue

**Affiliations:** 1 Institute of Mechanics and Engineering, Liaoning Technical University, Fu Xin, 123000 Liaoning China; 2 Petro China Research Institute of Petroleum Exploration and Development, Beijing, 100083 China

**Keywords:** Civil engineering, Energy infrastructure

## Abstract

Injection-production operation parameters, the minimum injection gas pressure (IGP: operation pressure), IGP interval, minimum IGP residence time, and injection-production cycle of the underground salt rock gas storage under long-term operation, affect not only the capacity and working ability of a salt cavern but also be crucial to the safety and stability of the surrounding rock. A 3D geo-mechanical model of the salt cavern is established to study the stability of the storage in the operation period. The deformation, expansion safety factor, volume shrinkage, and plastic zone are comprehensively considered for predicting the feasibility and stability of the salt cavern. The stability of the surrounding rock of the cavern with different injection-production parameters and the impact of each parameter on the stability of the cavern during operating are systematically investigated. The results indicate that the displacement, the expansion coefficient of the surrounding rock, and the volume shrinkage rate of the salt cavern reduces significantly with the increment of IGP interval and minimum IGP, while they increases with raising the minimum IGP residence time and injection-production cycle. With the continuous operation of the cavity, the displacement and the volume shrinkage rate enhances significantly year by year with the augment of operation parameters, moreover, their value show a fluctuating upward trend with the alternation of the gas injection and the production. The volume of the plastic zone is enlarged with the increment of the IGP interval, minimum IGP, and injection-production cycle, while it reduces with the extension of the minimum IGP residence time. The variation becomes remarkably with the augment of parameters. The sensitivity coefficients of each injection-production operation parameter are ranked, from large to small, as follows: IGP interval, minimum IGP, minimum IGP residence time, injection-production cycle. The results can offer beneficial reference for effectively optimize the injection-production parameters, so as to provide technical guidance for ensuring the stability of the storage and meeting requirements for the storage capacity.

## Introduction

**Figure 1:**
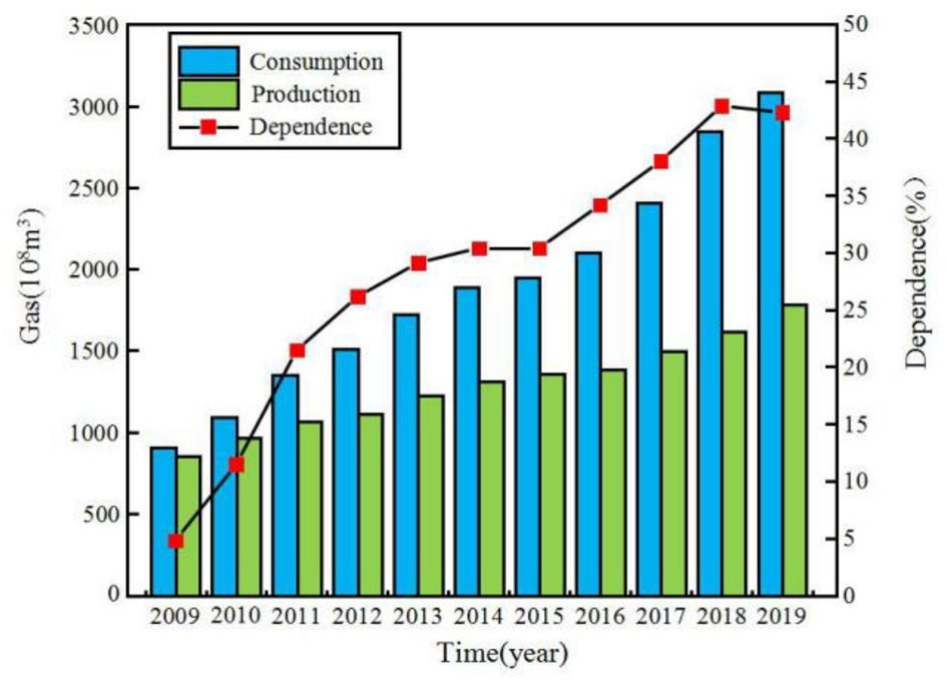
The consumption, production and dependence of natural gas in China^10^.

**Figure 2:**
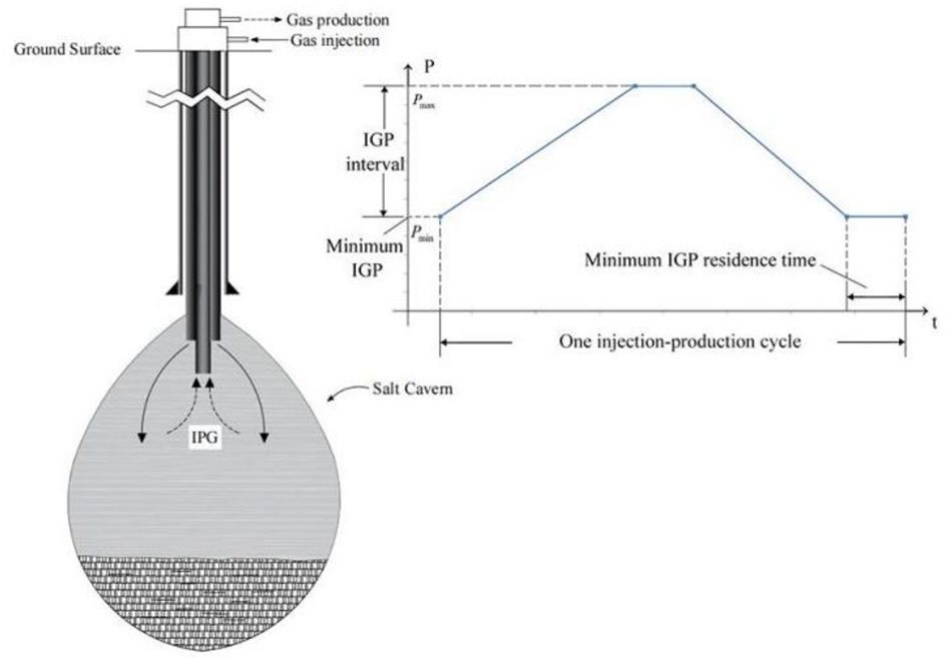
Salt cavern injection-production cycle and parameter diagram.

In recent years, the emergencies and the local war occur frequently in the world, and China’s dependence on the foreign natural gas has gradually increased (Fig. 1), which making the energy security situation increasingly serious^1^. Due to the excellent creep properties, low permeability, plastic deformation capacity, and recovery performance of the salt rock, the salt cavern has become an ideal place for deep underground energy storage^2–4^. Therefore nearly 100 deep underground salt rock storages have been built and put into operation world-wide. However, due to the long-term operation of the foreign salt cavern in the past three decades, the creep of salt rock, which is affected by the improper internal pressure control, has caused catastrophic accidents as fires and explosions, cavern failure, and surface subsidence. Such accidents can be sudden and destructive, and posing a large danger to the safety and the environment (Table 1)^5–9^. For example, in the 1990s, the Stratton Ridge salt cavern in Texas was collapsed due to the excessive creep of the salt rock, which created unsafe conditions for the injection-production process^7^. In 2001, the Yaggy salt cavern in Kansas collapsed during the gas injection-production, which is caused by the oil and gas leakage, and hundreds of people lost their lives due to fires and explosions^8^. According to the investigations, the reasons of the above accidents are closely related to the unreasonable injection-production parameters; the controllable parameters of the salt cavern include injection gas pressure (IGP) interval, minimum IGP, minimum IGP residence time, injection-production cycle, etc. (Fig. 2) (1) IGP interval can be expressed as $\left[ {P_{\min } ,P_{\max } } \right]$, where $P_{\max }$ and $P_{\min }$ are the maximum and the minimum value of the IGP. The IGP interval increases means $P_{\max }$ and $P_{\min }$ increase simultaneously, while the internal pressure difference $\Delta P$, $\Delta P = P_{\max } - P_{\min }$, remains unchanged. (2) Minimum IGP represents the minimum injection-production pressure ($P_{\min }$). The increase in the minimum IGP reduces the $\Delta P$, and the change of the $\Delta P$ may affect the cavern capacity and stability. (3) Minimum IGP residence time is the duration of maintaining the minimum pressure as constant. (4) One complete injection-production operation cycle is calculated with 1 step-up gas injection, 1 step-down gas recovery, and 2 stable pressure shut-ins. The number of injection-production cycle refers to the cyclic times for a complete injection-production cycle within one year. Furthermore, since the demand for the natural gas is related to the seasonal changes, the low-pressure residence time and the injection-production cycle should be adjusted accordingly. The rational allocation of parameters can not only enhance the storage and operational capacity of the salt cavern but also reduce the occurrence of disasters. Thus, the study of injection-production operation parameters is of great significance to the stability of the layered salt rock gas storage.

**Table 1:** Typical salt cavern accidents.

Name of storage cavern	Time and place of accident	Accident description	Accident cause	Economic loss	Influence scope
Kiel^5^	1967, Germany	12.3% loss in volume after 45 days	Excessive creep of salt	Cavern failure	The cavern
Eminence^5^	1970–1972, Mississippi, USA	More than 40% loss of volume	Excessive creep of salt	Cavern failure	The cavern
Tersanne^6^	1970–1980, France	Effective volume loss 35%, settlement rate 40 mm/a	Excessive creep of salt	Cavern deactivated	Influence range approximately 2000 m
Stratton Ridge^7^	1990s, Texas, USA	Cavern abandoned, ground subsidence, settlement rate 40 mm/a	Excessive creep of salt and in wet condition	Cavern failure	Ground above the caverns
Yaggy^8^	2001, Kansas, USA	Fire and explosion	Failure and damage of casing during gas injection	About 5600,000 m^3^ natural gas loss	Part of the town affected, hundreds of people evacuated
Moss Bluff^9^	2004, Texas, USA	Fire and explosion	Brine pipe corrosion	At least 36 million US $ loss	Influence range was 120 m, people within 5 km evacuated

Up till now, many achievements have been made in evaluating the stability of the layered salt rock gas storage. In 1989, Schmidt et al.^11^ identified the minimum internal operation pressure of the salt cavern, and proposed that the cavern volume was decreased after the long-term operation and the cavern would eventually become unusable. In 2002, Bruno et al.^12^ discussed a method to determine the ultimate operation pressure of salt cavern. The sidewall and roof of the layered salt rock gas storage may collapse during operation, and the ultimate operation pressure of the storage depends on the geological conditions and the mechanical properties of the salt rock. In 2004, Liu et al.^13^ investigated the operation pressure of the gas storage in a thin salt layer by analytical method and numerical analysis. In 2005, Yang et al.^14^ established a Cosserat medium expansion constitutive model by considering the meso-bending effect in the macro-average sense. Besides, numerical simulations were carried out under different internal pressures, and then the corresponding optimal operation pressure was obtained. In 2006, Chen et al.^15^ carried out a numerical model of Jintan salt cavern to study the creep deformation and plastic damage zone of salt rock, and proposed an acceptable internal gas operation pressure and casing shoe depth. In 2008, Liang et al.^16^ obtained the ultimate operation pressure by studying the physical and mechanical properties of the layered salt rock gas storage. Cao et al.^17^ established an injection-production dynamic model of the underground salt cavern, and the operation parameters was determined by analyse the operation characteristics of cyclic injection-production. In 2012, Zhang et al.^18^ studied the influence of the gas injection-production rate, operation pressure, and pillars width on the operation safety and stability of the layered salt rock storage system through a 3D rheological geological model. Ji et al.^19^ reported the deformation law of the underground salt cavern under different injection-production schemes for 30 years. The low-pressure operation period, which is after the emergency gas extraction, was the main stage of the volume convergence of the underground salt cavern. In 2014, Zhang et al.^20^ argued the long-term stability of the underground salt cavern with interlayers under different internal pressure conditions. The influence of the operation pressure on the shrinkage of the salt cavern was greater than that of the interlayers. In 2016, Wang et al.^21^ investigated the minimum internal operation pressure of the multilayer gas storage. They proposed that increasing the span and depth of the top of the salt cavern can increase the minimum allowable operation pressure. The increase in the elastic modulus of the adjacent interlayer at the top of the salt cavern leads to a decrease in the minimum allowable pressure. In 2017, Ma et al.^22^ established underground salt cavern models, which have different internal operation pressures and different ratios of height to diameter, to determine the optimal value of operation parameters. In 2018, Wang et al.^23^ developed a 3D geo-mechanical model for JK-A salt cavern to study the stability of the roof of the storage. They postulate that the roof collapse of the cavern is related to the operation pressure and the rate of pressure decrease. Zhang et al.^24^ proposed a method for determining the upper and lower limit of the operation pressure. In 2019, Liu et al.^25^ studied the stress of the cavern wall under different injection-production rates. It was concluded that the rapid injection-production leads to a tensile stress increasing, and tensile stress zones exist clearly at the top and bottom of the cavern. Wang et al.^26^ established a 3D geo-mechanical model based on the sonar data of Jintan salt cavern combined with the characteristics of the target stratum. Based on the international standards, the King-1 cavern was safe when the maximum operation pressure enhanced from 17 MPa to 18 MPa. In 2020, the thermomechanical model, which is proposed by Li, provided a method for evaluating the long-term stability of underground rock salt caverns, and it was used to determine the optimal operation parameters and salt cavern design^27^. Makhmutov et al.^28^ used a 2D finite element model with unstructured grids to model and simulate the complex creep behaviour of rock salt caves, which can be employed to evaluate the long-term safety and reliability of the roof structure. Moreover, a sensitivity analysis of key parameters was carried out.

So far, a number of researchers have reported the impact of IGP on the stability of the salt cavern. However, there has been little discussion about the rational allocation and the influence extent of the gas injection-production operation parameters. The objectives of this research are to explore the effect of the minimum IGP, IGP interval, minimum IGP residence time, and injection-production cycle on the surrounding rock of the salt cavern. The deformation, expansion safety factor, volume shrinkage, and plastic zone are analysed, and the sensitivity of the safety and stability of the salt cavern to the operation parameters are obtained. The findings can provide a theoretical basis and technical guidance for the operation internal pressure design of the salt cavern.

## Safety and stability evaluation index of salt cavern

To evaluate the stability of the salt cavern during operation, the surrounding rock deformation, cavern volume shrinkage, expansion safety factor, and plastic zone volume are introduced and comprehensively counted as the stability indices to evaluate the safety of the salt cavern (Table 2). These evaluation indices considered the impact of the creep, shear, tension, expansion, and shrinkage on the salt cavern during operation. They are widely used for the stability evaluation of the salt cavern.

**Table 2:** Evaluation index of salt cavern stability.

Index	Author	Year	Criterion
Displacement	Yang et al.^29^	2009	The maximum displacement of caverns should not exceed 5% of the maximum cavern diameter
	Zhang et al.^30^	2017	The maximum displacement of single-well-vertical (SWV) caverns should not exceed 10% of the maximum cavern diameter
	Wang et al.^26^	2018	The maximum displacement of single-well-vertical (SWV) caverns should not exceed 5% of the maximum cavern diameter
	Chen et al.^31^	2020	The maximum displacement of small-spacing two-well (SSTW) should not exceed 5% of the maximum cavern diameter
	Li et al.^10^	2021	The maximum displacement of U-shaped horizontal (UHSC) caverns should not exceed 7% of the maximum cavern diameter
Dilatancy safety factor	Spiers et al.^32^	1989	$\sqrt {J_{2} } = 0.27I{}_{1} + 1.9$
	Ratigan et al.^33^	1991	$\sqrt {J_{2} } = 0.27I{}_{1}$
	Hunsche^34^	1993	$\sqrt {J_{2} } = - 2.286 \times 10^{3} \times I_{1}^{2} + 0.351 \times I_{1}$
	Spiers et al.^32^	2004	$\sqrt {J_{2} } = 12.04 - 9.104e^{{ - {0}{\text{.04931}}I_{1} }}$
	Alkan et al.^35^	2007	$\sqrt {J_{2} } = \frac{{0.54I{}_{1}}}{{1 + 0.013I_{1} }}$
	Labaune and Rouabhi^36^	2018	$\sqrt {J_{2} } = 0.25I{}_{1} + 1.44$
Volume shrinkage	Bérest and Minh^37^	1981	30-year volume shrinkage of salt cavern ≤ 30%
	Hou and Wu^38^	2003	30-year volume shrinkage of salt cavern ≤ 20%
	Brouard et al.^39^	2012	1-year volume shrinkage ≤ 1%, 30-year volume shrinkage ≤ 30%
	Liu et al.^40^	2018	1-year volume shrinkage ≤ 1%, 5-year volume shrinkage ≤ 5%, 30-year volume shrinkage ≤ 30%
	Chen et al.^31^	2020	1-year volume shrinkage ≤ 1%, 30-year volume shrinkage ≤ 30%
Plastic zone	Wang et al.^41^	2015	$f^{s} = \sigma_{1} - \frac{1 + \sin \varphi }{{1 - \sin \varphi }}\sigma_{3} - \frac{2c \cdot \cos }{{1 - \sin \varphi }}$, $f^{t} = \sigma_{t} - \sigma_{3}$
	Ma et al.^42^	2015	$f^{s} = \sigma_{1} - \frac{1 + \sin \varphi }{{1 - \sin \varphi }}\sigma_{3} - \frac{2c \cdot \cos }{{1 - \sin \varphi }}$, $f^{t} = \sigma_{t} - \sigma_{3}$
	Yang et al.^43^	2016	$\frac{1}{2}(\sigma_{1} - \sigma_{3} ) = C\cos \varphi - \frac{1}{2}(\sigma_{1} + \sigma_{3} )\sin \varphi$
	Zhang et al.^30^	2017	$\frac{1}{2}(\sigma_{1} - \sigma_{3} ) = C\cos \varphi - \frac{1}{2}(\sigma_{1} + \sigma_{3} )\sin \varphi$
	Liu et al.^44^	2020	$f^{s} = \sigma_{1} - \frac{1 + \sin \varphi }{{1 - \sin \varphi }}\sigma_{3} - \frac{2c \cdot \cos }{{1 - \sin \varphi }}$, $f^{t} = \sigma_{t} - \sigma_{3}$

### Surrounding rock deformation

Surrounding rock deformation, especially roof subsidence, is an important index for reflecting the stability of the salt cavern and easy to monitor in simulations^26,29,31^. Based on the distribution of surrounding rock displacement, the deformation characteristics at each position of the salt cavern can be clearly obtained. Moreover, the maximum displacement of the surrounding rock of the salt cavern should meet the following criteria:

1 $$D_{\max } \le 5{\text{\% }}d_{\max }$$

where *D*_max_ is the maximum displacement and *d*_max_ is the maximum salt cavern diameter.

### Expansion safety factor

**Figure 3:**
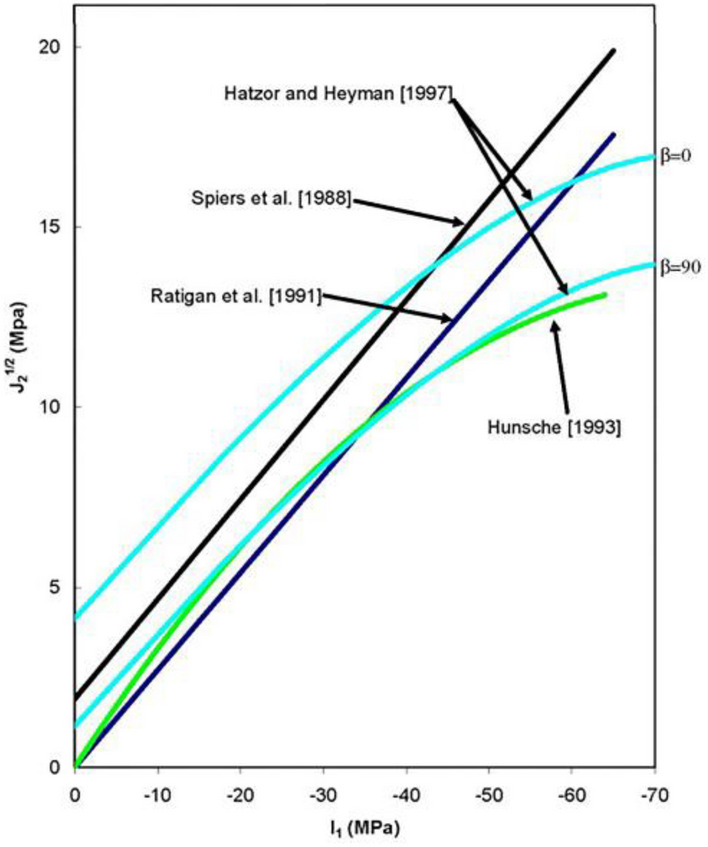
The evolving graph of the expansion failure criterion of salt rock^33^.

The expansion safety factor is an important indicator of many practical engineering applications. When the rock exists in a complex stress state, the expansion failure may impact the sealing capacity of the storage and cause leakage of the gas. Therefore, the damage due to the expansion of salt rock must be avoided during the long-term injection and production operation of the salt cavern. Referring to the research of Spiers et al.^32^, Ratigan et al.^33^, and Hunsche et al.^34^ (Fig. 3), the expansion failure criterion of the salt rock is established as follows:

2 $$SF = \frac{{\sqrt {J_{2} } }}{{aI_{1} + b}} \ge 1$$

where *SF* is the safety factor; *a* and *b* are the coefficients of expansion, subject to test fitting (different researchers have obtained different expansion coefficients according to the test, thus showing different fitting curves in Fig. 3); $I_{1}$ is the first stress invariant; and $J_{2}$ is the second stress deviation invariant. $I_{1}$ and $J_{2}$ can be calculated by Eqs. (3) and (4):

3 $$I_{1} = \sigma_{1} + \sigma_{2} + \sigma_{3}$$

4 $$J_{2} = \frac{1}{6}\left[ {(\sigma_{1} - \sigma_{2} )^{2} + (\sigma_{2} - \sigma_{3} )^{2} + (\sigma_{3} - \sigma_{1} )^{2} } \right]$$

where $\sigma_{1}$, $\sigma_{2}$ and $\sigma_{3}$ are the maximum, intermediate, and minimum principal stresses, respectively.

### Volume shrinkage of the cavern

Salt cavern volume shrinkage is an important index to evaluate the availability and economy of the salt cavern. It is defined as the ratio of the volume reduction to the original volume of the salt cavern. According to the reference^39^, in China, the volume shrinkage rate of the salt cavern should satisfy the following:

5 $$\frac{{V - V_{t} }}{V} \times 100\% \le 20{\text{\% }}$$

where *V* is the original volume of the salt cavern, and *V*_*t*_ is the current volume of the salt cavern.

### Plastic zone volume

The failure modes of the salt rock mainly include shear failure and tensile failure. The plastic failure of the rock mass around the salt cavern is determined by criterions within FLAC^3D^ software, the Mohr‒Coulomb criterion (6) and the maximum tensile stress criterion (7). Many publications use this criterion as an indicator, and it has proven to be accurate and reliable^29^.

6 $$f^{s} = \sigma _{1} - \frac{{1 + \sin \varphi }}{{1 - \sin \varphi }}\sigma _{3} - \frac{{2c \cdot \cos \varphi }}{{1 - \sin \varphi }}$$

7 $$f^{t} = \sigma_{t} - \sigma_{3}$$

where $\sigma_{1}$ is the maximum principal stress, $\sigma_{3}$ is the minimum principal stress, *c* is the cohesion, $\varphi$ is the internal friction angle, and $\sigma_{t}$ is the tensile strength of the rock mass.

**Figure 4:**
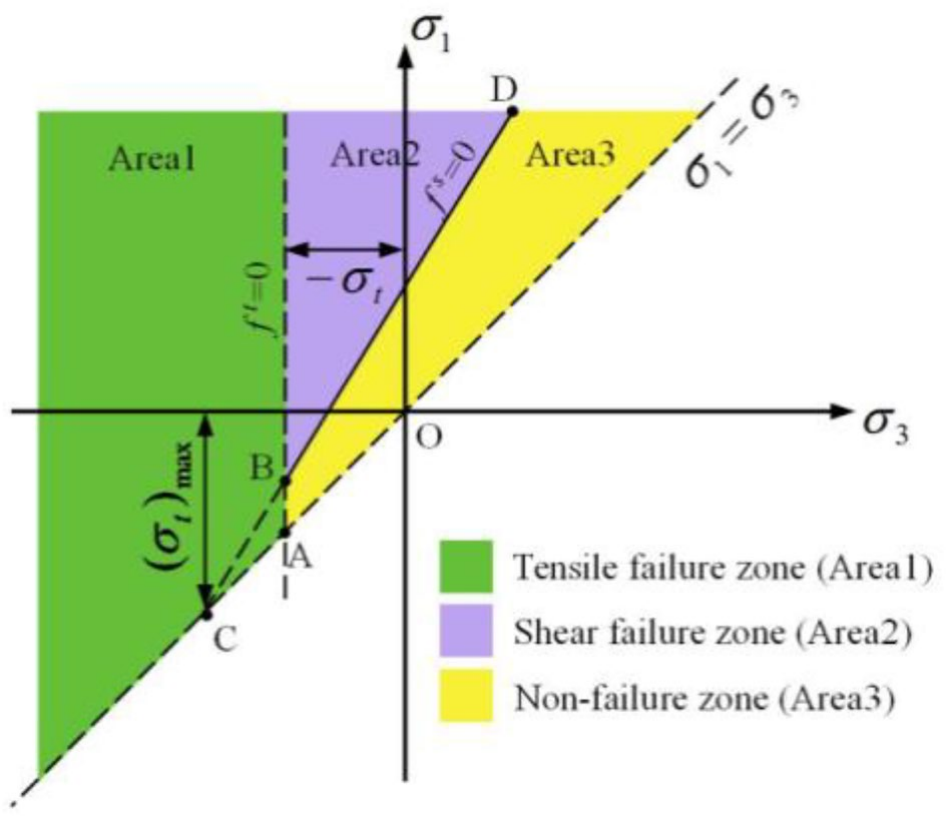
Mohr‒Coulomb failure criterion^42^.

As shown in Fig. 4, the failure criterion in FLAC^3D^ divides the stress space into three areas. Area 1 is the tensile failure zone, Area 2 is the shear failure zone, and Area 3 is the non-failure zone. The volume of the plastic zone is equal to the sum of the shear failure zone and tensile failure zone.

- When $\sigma_{3} > \sigma_{t}$, if the shear failure function $f^{s} > 0$, the stress state is located in Area 2. Otherwise, the stress state is located in Area 1, and no damage will occur.
- When $\sigma_{3} < \sigma_{t}$, the stress state is located in Area 3, and tensile failure occurs.

## Numerical simulation

### 3D geo-mechanical model and its boundary conditions

**Figure 5:**
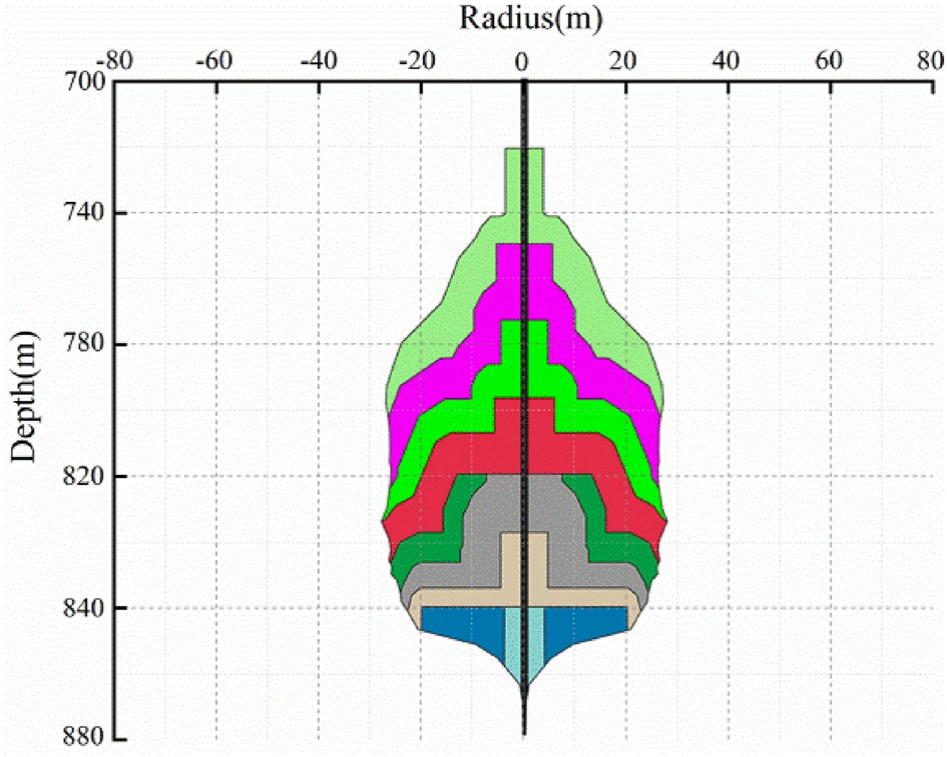
The shape of the cavern after water solution.

**Figure 6:**
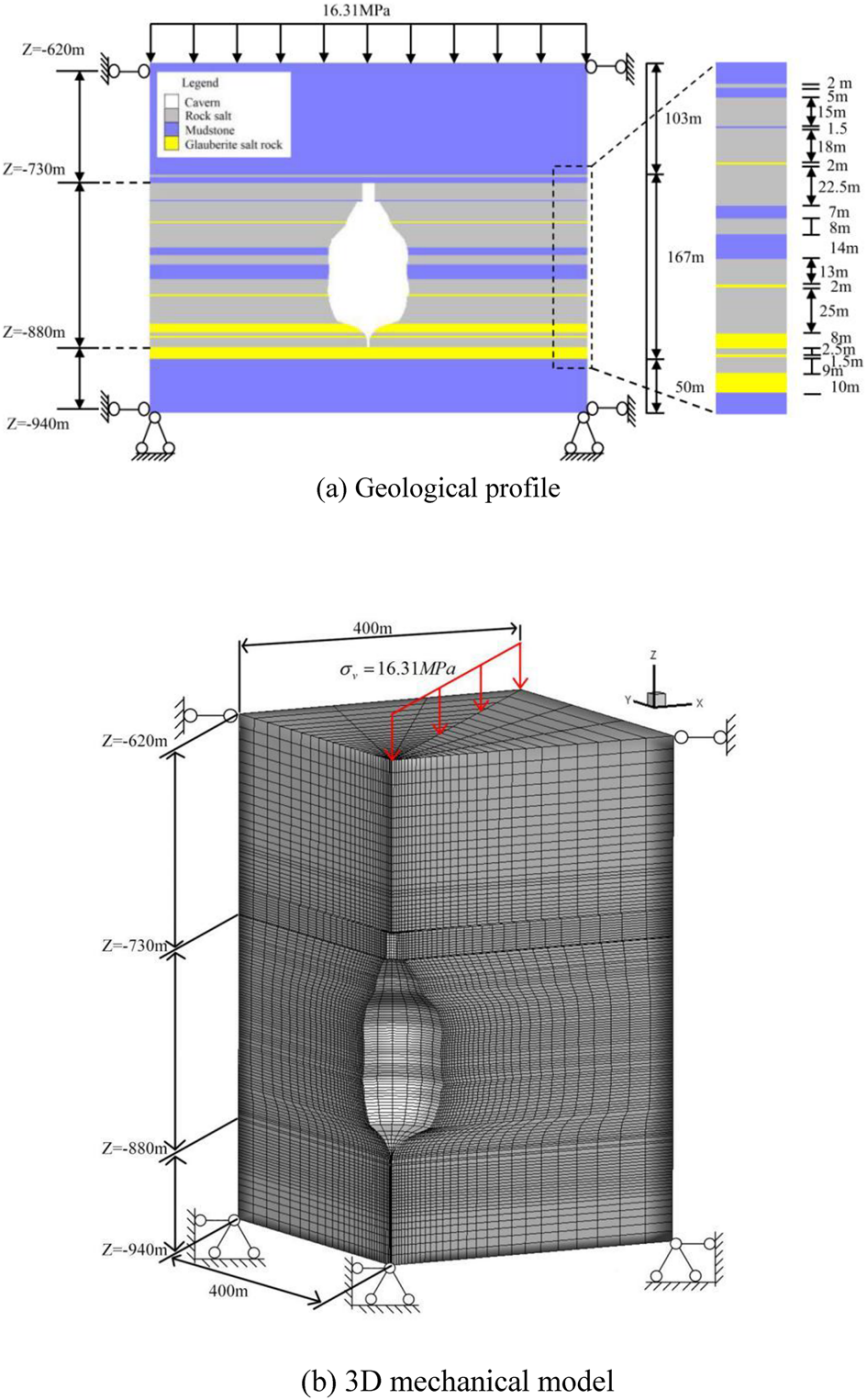
3D geo-mechanical model.

To study the effect of injection-production operation parameters on the salt cavern during operation period, taking one proposed layered salt rock underground gas storage as the engineering background. The shape of the cavern after water solution is obtained via WinUbro (Fig. 5), based on the complete investigation of the actual geological conditions. The interlayers, with the thickness ranging from 1 m to 14 m, unevenly distributed in the rock salt formation. The height, maximum diameter, buried depth (The distance from the ground to the top of the cavity), and effective volume of the cavern are 150 m, 76 m, 730 m, and 33.6 × 10^4^ m^3^, respectively. A 3D axisymmetric mechanical cube model is established via FLAC^3D ^(Fig. 6). The vertical direction is defined as the Z axes, and the positive direction of the axes is upward. The depth of the coordinate origin of the 3D model is 940 m, and the XY plane size is 800 m × 800 m. The weight of the overlying rock is set as the upper surface load on the model, and the equivalent load is calculated according to the actual thickness $h$ (620 m) and average density $\rho$ (2.63 × 10^3^ kg/m^3^) of the formation. The average gravity calculation formula is shown as Eq. (8).

8 $$\sigma_{z} = \rho gh$$

where *g* is the acceleration due to gravity; $\sigma_{z}$ is the overburden pressure; $h$ is the height of overlying strata.

The fixed condition is imposed at the bottom of the model, and the normal constraints boundary condition is imposed at the surrounding.

### Design of the gas injection-production operation scheme

According to the documents “Design Specification of Salt Rock Gas Storage Cavern” and “Safety Specification For Salt Rock Underground Gas Storage”^45,46^ and the logging data, the upper limit pressure should not exceed 80% of the overlying strata pressure and fracture pressure. The maximum operation pressure of the salt cavern is designed to be 13 MPa according to the weight of the cavern roof. The minimum operation pressure of salt cavern is determined to be 4 MPa by referring to the pressure gradient, which is 0.7 MPa/100 m, of the lower limit pressure of the Jintan salt cavern, combine with the gas recovery capacity, the wellhead pressure, the salt cavern stability, and other factors. Therefore, the IGP range is 4 MPa–13 MPa.

**Figure 7:**
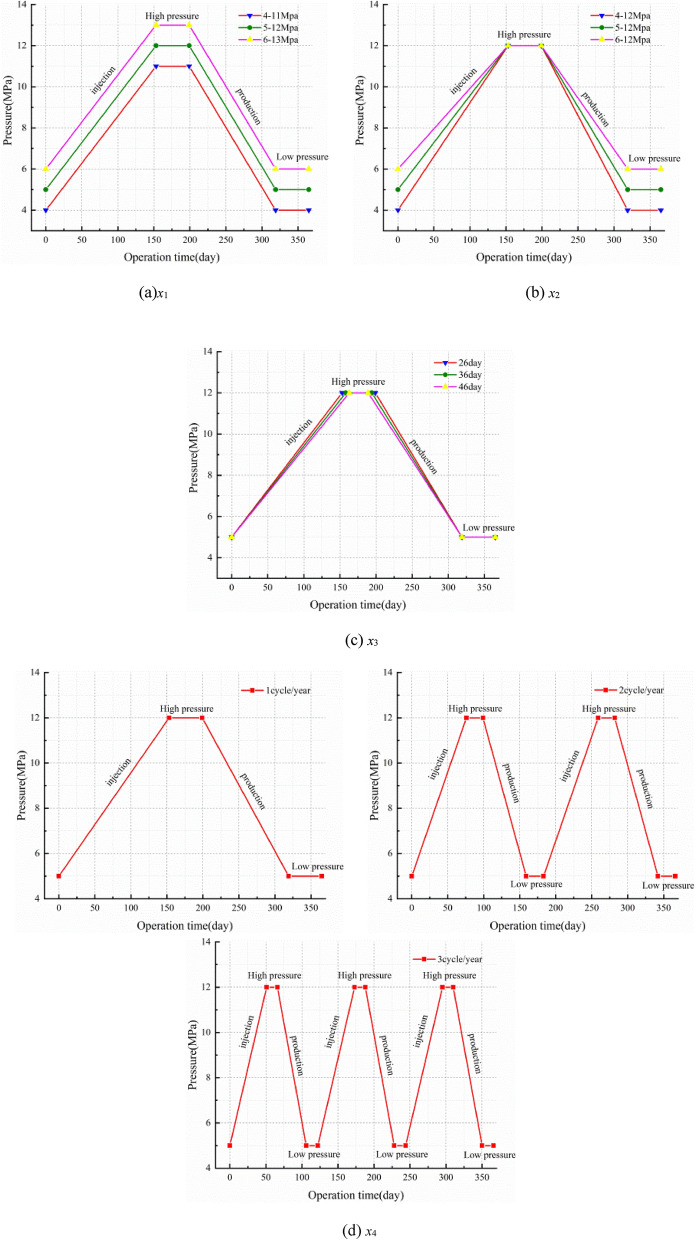
Operation pressure of the salt cavern.

To research the stability of the rock mass around the cavern under different injection-production operation parameters, a numerical calculation scheme is designed as shown in Table 3 through referring to China's existing Jintan salt cavern operation scheme^47,48^ and China's market demand^49^. The scheme considered four influencing factors $x_{i} \left( {i = 1,2,3,4} \right)$, and each factor corresponds to three cases a, b and c. The duration of the injection-production process is stable for 30 years, and the variation of the operation pressure in different cases for one year is shown in Fig. 7. The detailed simulation scheme is as follows:

- *x*_1_: Fig. 7a shows the operation pressure which obtained with considering the IGP interval increase. The designed IGP ranges are 4–11 MPa, 5–12 MPa, and 6–13 MPa.
- *x*_2_: Fig. 7b shows the operation pressure which obtained with considering an unchanged upper limit of IGP and an increase in the lower limit of IGP. The designed IGP ranges are 4-12 MPa, 5-12 MPa, and 6-12 MPa.
- *x*_3_: Fig. 7c shows the operation pressure which obtained with considering different residence time of the minimum IGP as 26 days, 36 days, and 46 days.
- *x*_4_: Fig. 7d shows the operation pressure which obtained with considering the designed injection-production cycle as once a year, twice a year, and three times a year.

**Table 3:** Comparison scheme design of various influencing parameters.

Simulation number	Case a (benchmark scheme)	Case b	Case c
*x*_1_ (IGP range; MPa)	5–12	4–11	6–13
*x*_2_ (Minimum operation IGP; MPa)	5–12	4–12	6–12
*x*_3_ (Dwell time of the minimum IGP; day/year)	46	26	36
*x*_4_ (Cycle IGP; cycles/year)	1	2	3

### Select calculation parameters

The salt layer in the reservoir area is mainly composed of salt rock, salt-bearing calcium mirabilite interlayers, and mudstone. Rock mechanical-parameters, which are shown in Table 4, are determined by the routine laboratory creep tests. The steady-state creep rate obeys the Norton energy law, and the standard form of the Norton exponential model is shown in Eq. (9).

9 $$\dot{\varepsilon }(t) = Aq^{n}$$

where $\dot{\varepsilon }(t)$ is the steady-state creep rate; *J*_2_ in $q = \sqrt {3J_{2} }$ is the second invariant of the deviator stress; *A* is the material characteristic parameter; and *n* is the constant of the stress index.

**Table 4:** Rock mechanical parameters.

Property	Material		
	Rock salt	Mudstone	Interlayer
Young’s modulus (GPa)	6.84	22.9	38.5
Poisson’s ratio	0.21	0.15	0.20
Cohesion (MPa)	8.40	7.75	5.80
Friction angle (°)	39.3	45.4	46.9
Tensile strength (MPa)	1.38	3.19	1.42
A (creep model) (MPa^-*n*^ h^−1^)	9.0 × 10^–7^	2.8 × 10^–6^	2.8 × 10^–6^
n (creep model)	2.62	2.10	2.10

## Results and discussion

**Figure 8:**
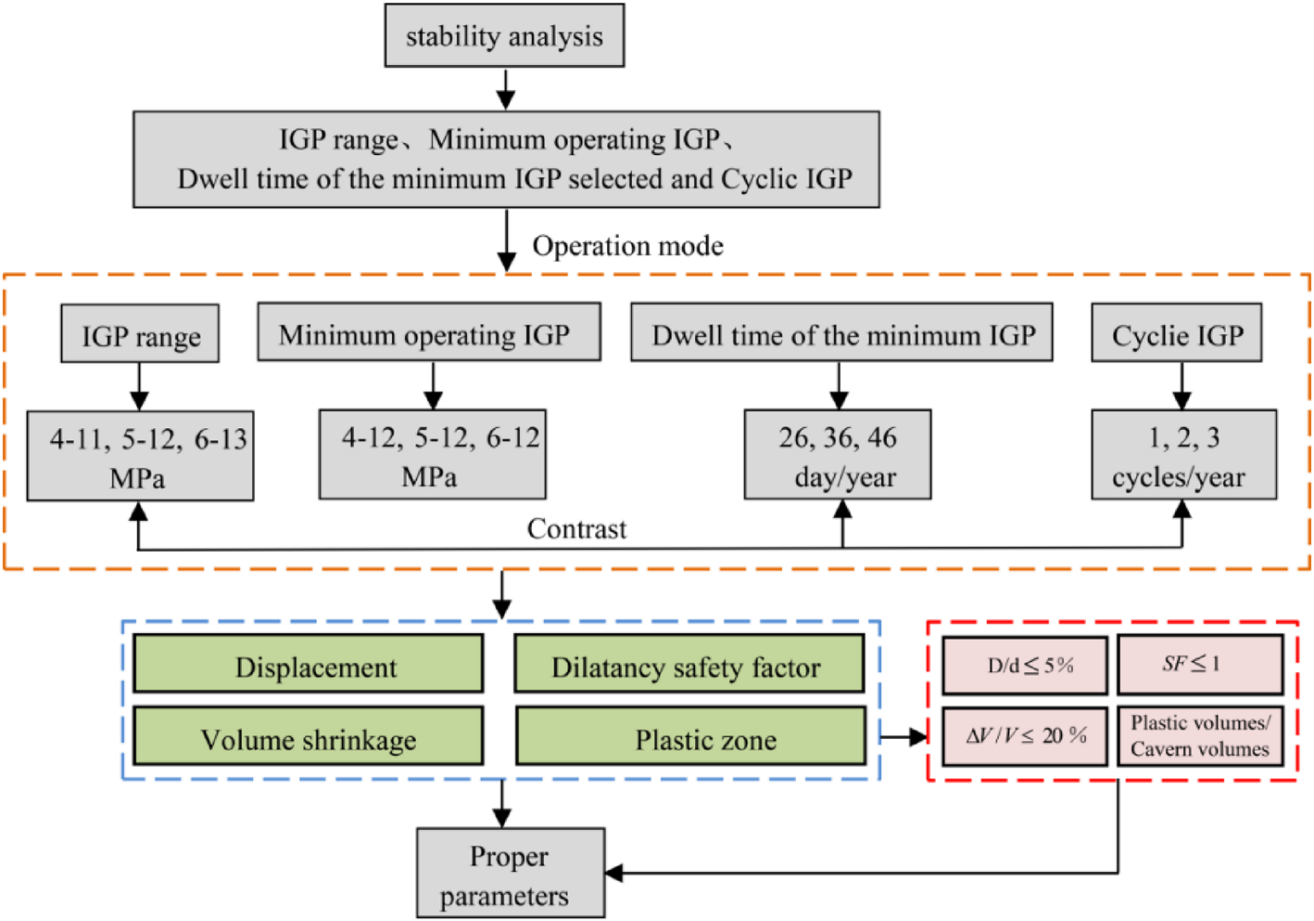
Salt cavern stability assessment.

The numerical simulation for the mechanical behaviour of the surrounding rock after 30 years of operation is performed. The stability indices are introduced to evaluate the safety of the salt cavern, and the deformation, expansion failure, volume shrinkage, and plastic zone are comparatively compared and analysed. Through sensitivity analysis of different injection-production operation parameters (IGP interval, minimum IGP, minimum IGP residence time, and injection-production cycle), the main factors of deformation and failure of the surrounding rock is find out. The flow chart of the salt cavern stability assessment and the selected injection-production operation parameters are shown in Fig. 8.

### Deformation law of surrounding rock in salt cavern

**Figure 9:**
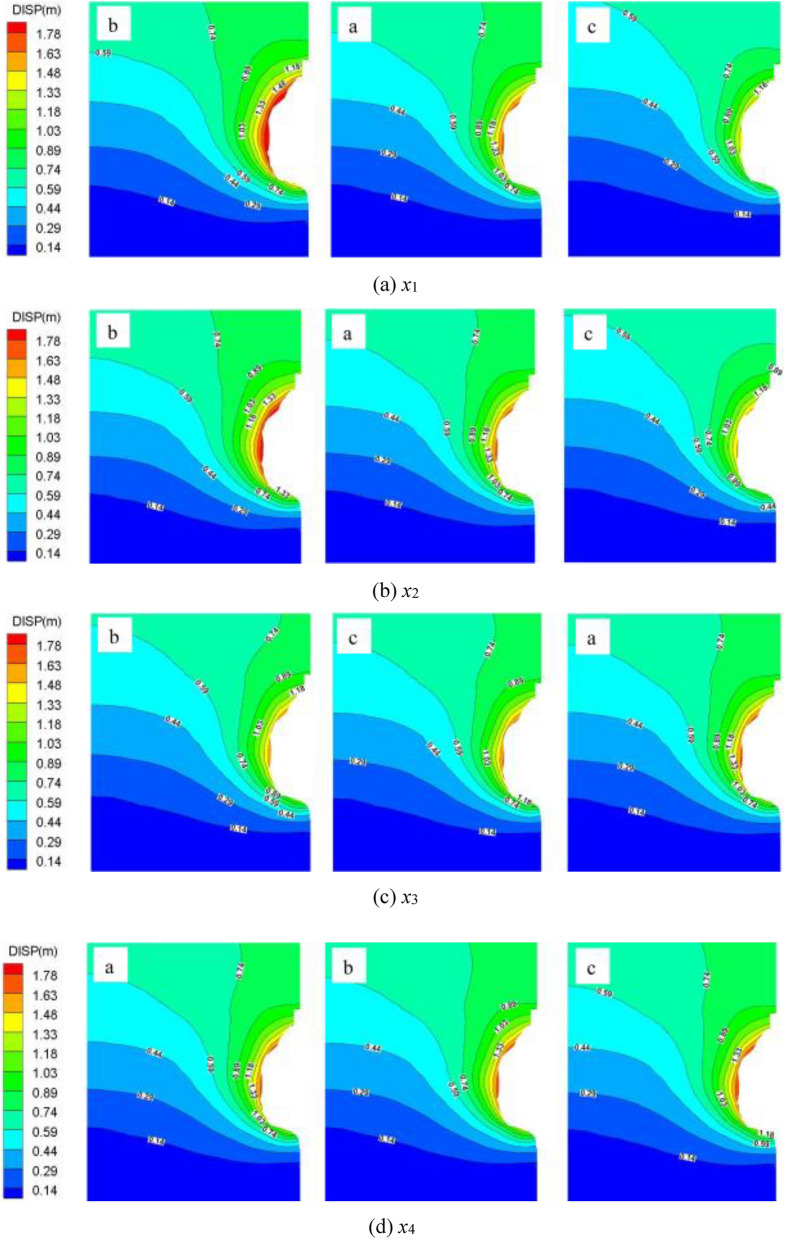
Total deformation contours under different schemes after 30 years of operation.

Figure 9 presents the contours of the surrounding rock deformation of the salt cavern after the cyclic injection-production by different schemes. As shown in Fig. 9, the distribution of the displacement, from the cavern wall to the boundary of the model, changed from the contour around the cavern to the layered formation after 30 years of operation. Moreover, it presents a gradient change, and the displacement changed significantly at the near-field of the cavern wall. It decreases with the distance away from the cavern wall and exhibit differently at each point of the cavern wall. The maximum values show significant diversity under different operation conditions, and it appears at the roof of the cavern. In scheme X1, X2, X3, and X4, the maximum displacement (1.78 m, 1.70 m, 1.65 m, and 1.60 m) are 2.28%, 2.17%, 2.11%, and 2.05%, respectively, of the maximum diameter of the salt cavern. The displacement of the surrounding rock decreases significantly with the augmentation of the IGP interval and minimum IGP, but it is enhanced with increasing the minimum IGP residence time and injection-production cycle. While the IGP interval and the minimum IGP increase, the stress of the surrounding rock become close to that of the original rock that before a cavern was built, which may leads to a more stable cavern. However, the extension of the minimum IGP residence time, which means the storage stay in a low pressure for a longer time, will result in a longer duration of the creep deformation. In addition, the increment of the injection-production cycle number, which means the injection-production rate of the salt cavern is raised, will lead to a faster damage of the surrounding rock. The increase of the minimum IGP residence time or the cycle number will leads to a greater displacement of the surrounding rock.

**Figure 10:**
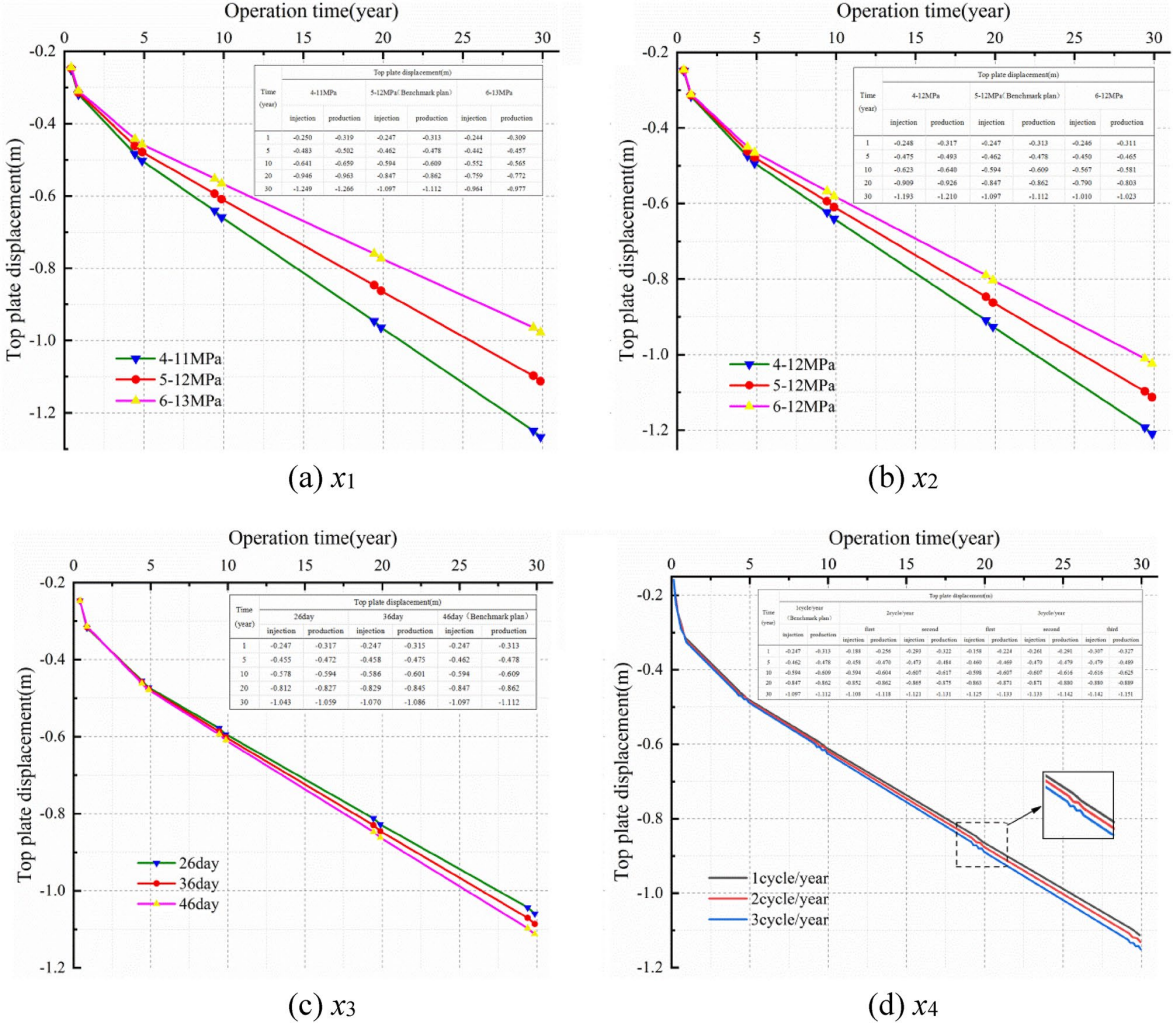
Line diagram of roof displacement‒time in different schemes.

Figure 10 plots the displacement at the roof of the cavern varied with time in different schemes. It can be seen from the Fig. 10 that the displacement augment with time in different schemes, meanwhile, the curve slope decreases year by year. The gas injection-production process was carried out in the salt cavern every year. Firstly, the displacement decreases gradually with the gas injection, and then it augments rapidly with the gas production. The displacement presents a cyclical fluctuation increase year by year, and the trend becomes obviously over time. This result may be explained by the fact that the increment of the internal pressure of the salt cavern during gas injection inhibits the shrinkage ability of the surrounding rock, while the decrease of the internal pressure during gas production enhances the shrinkage ability. And the shrinkage capacity is determined by the squeezing effect of the in-situ stress on the cavern.

The displacement difference between different cases is enlarged gradually with time under different schemes. In scheme X1, the displacement at the roof of the cavern, which obtained when the IGP range are set as 4–11 MPa and 6–13 MPa, are increased by 13.85% and 12.14%, respectively, compared with the result obtained when the range is 5–12 MPa (benchmark scheme) after 30 years operation. In the X2 scheme, the displacement at the cavern roof, which got when the minimum IGP are set as 4 MPa and 6 MPa, are increased by 8.81% and decreased by 8.0%, respectively, compared with the result obtained with the minimum IGP is 5 MPa (benchmark scheme). In the X3 scheme, when the low-pressure residence time are set as 26 days and 36 days, the displacement of the cavern roof are reduced by 4.77% and 2.34%, respectively, compared with the result obtained when the range is 46 days (benchmark scheme) after 30 years operation. In the X4 scheme, the displacement at the cavern roof, which obtained when the injection-production cycle are set as two and three cycles, are increased by 1.71% and 3.51%, respectively, compared with the result got when the cycle is one time in a year after 30 years operation.

Therefore, the displacement at the roof of the cavern decreases significantly with the enhancement of the IGP interval and the minimum IGP, while it is augmented with the increase of the minimum IGP residence time and injection-production cycle. And the variation tendency becomes more obvious over time.

### Expansion safety factor

**Figure 11:**
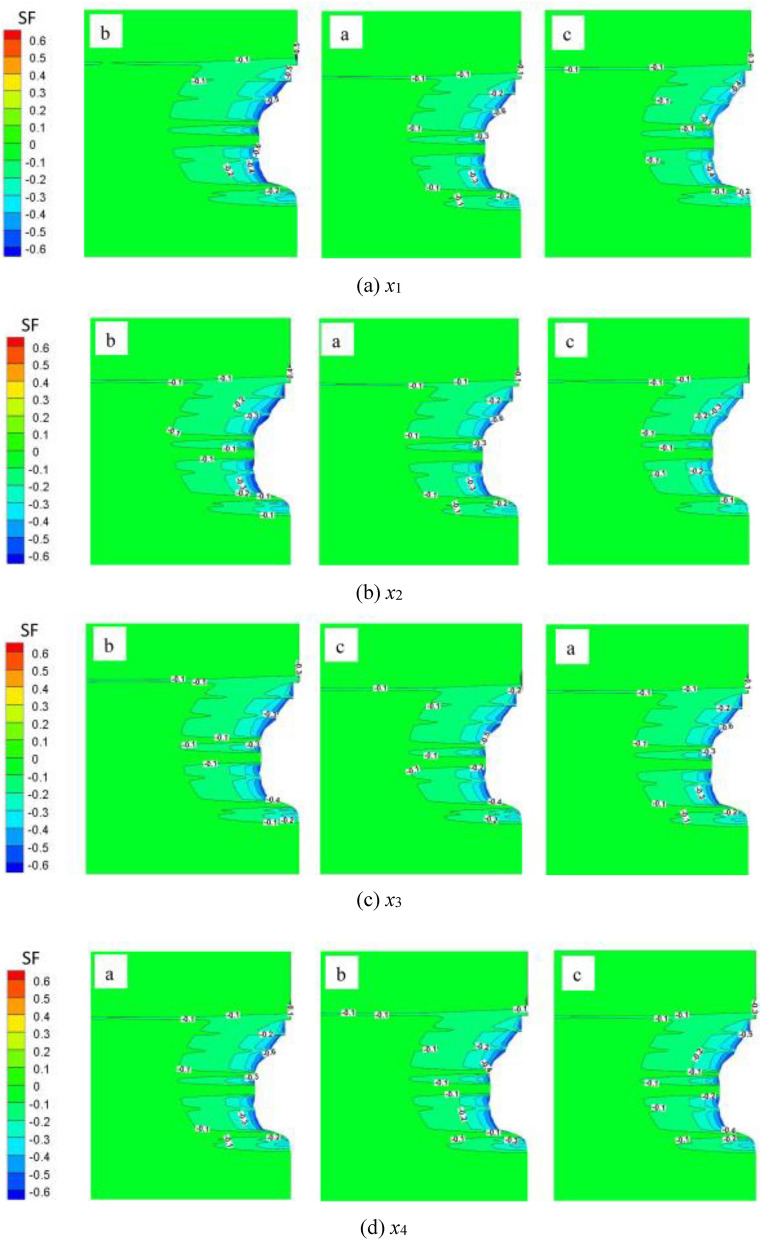
Safety factor (SF) isolines of salt cavern after 30 years of operation of different schemes (after gas production).

Using Eq. (2), the isoline of the expansion safety factor of the surrounding rock of the salt cavern under different schemes after 30 years is obtained. As is apparent in Fig. 11, all of the expansion coefficients are less than 1, which means no expansion failure phenomenon existed in the salt rock. The value of the safety factor at all position of the cavern wall is small, in which the absolute value of the interlayer was greater than that of the salt layer; the expansion damage is more likely to occur in the salt layer. The mainly reason is that the layered sedimentary structure of the interlayer has high strength, and its existence has an effective constraint on the salt rock, which is conducive to the stability of the salt cavern. The expansion coefficient of the surrounding rock increases significantly with enhancing the IGP range and the minimum IGP, while it decreases with the increment of the minimum IGP residence time and the injection-production cycle.

### Trend of salt cavern volume

**Figure 12:**
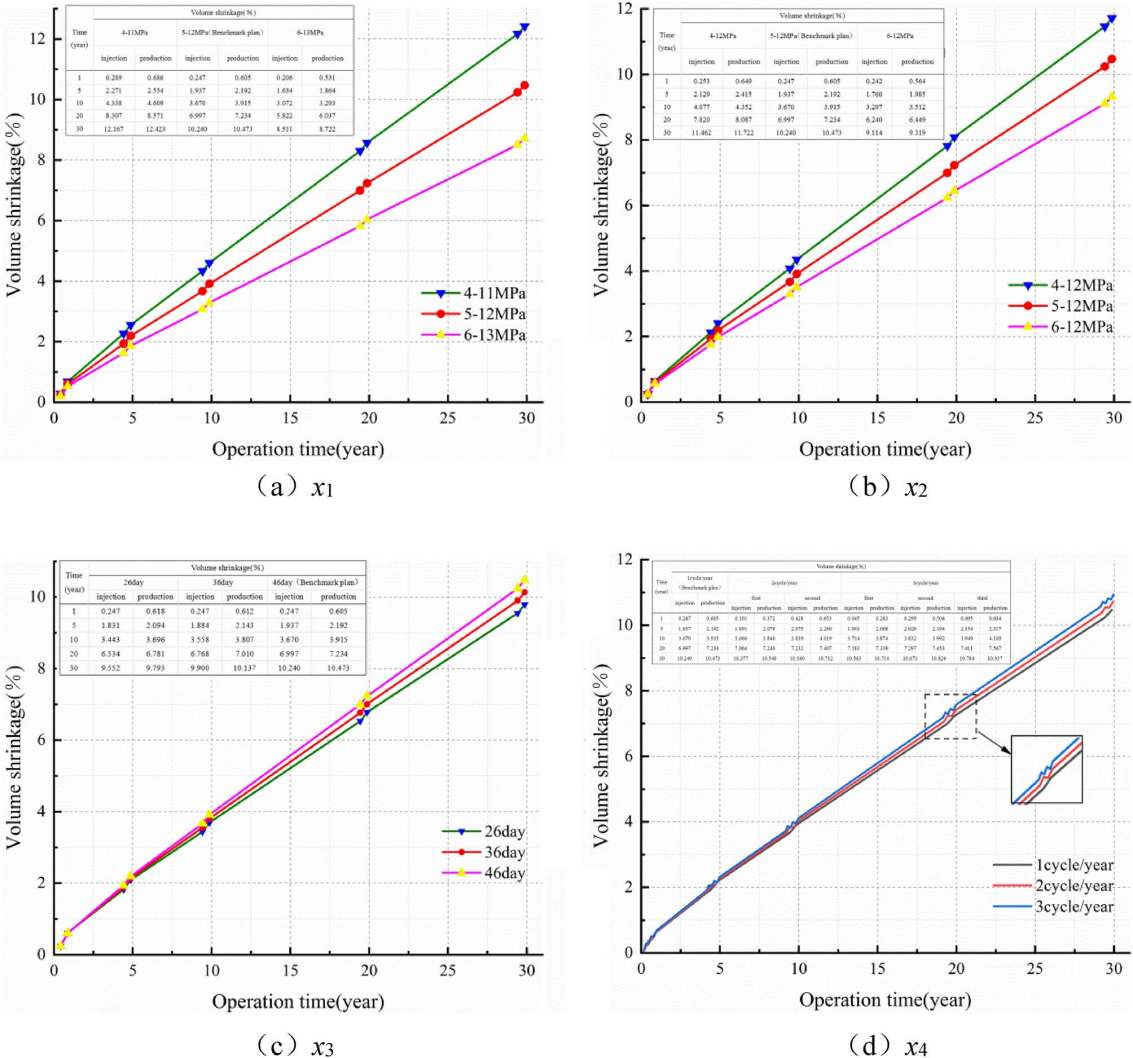
Line diagram of volume shrinkage rate with time in different schemes.

Figure 12 illustrates the volume shrinkage rate of the salt cavern in different schemes for 30 years. Form the Fig. 12 we can see that the volume shrinkage rate of the salt cavern augments with time, while the slope of the curve decreases year by year. Moreover, the differential value of the volume shrinkage between different cases in each scheme gradually becomes larger over time. Figure 12a, b show that the volume shrinkage rate of the salt cavern decreases significantly with enhancing the IGP interval and minimum IGP. The maximum volume shrinkage is 12.423% and 11.722% at 4–11 MPa and 4–12 MPa, respectively. Figure 12c, d show that the volume shrinkage rate augment with increasing the minimum IGP residence time and injection-production cycle. In scheme X3 and X4, the maximum volume shrinkage is 10.473% and 10.937%, respectively. The volume shrinkage, which has a similar regulation with the displacement at the cavern roof, presents a cyclical fluctuation increase year by year, and the increase trend becomes obviously over time. The dominant reason is that the limited pressure of the salt cavern in operation is usually lower than the original in-situ stress. So the volume of the storage becomes smaller due to the compression of the cavern that caused by the squeezing effect of the in-situ stress. Nevertheless, the volume shrinkage rate of the cavern does not exceed 20% under different schemes for 30 years operation.

### Plastic zone

The appearance of the plastic zone actually reflects the shear failure and tensile failure of the surrounding rock. These failures indicate that the unit has irreversible plastic deformation, which may cause the initiation and propagation of cracks. Therefore, to compare the effects of different injection-production parameters on the stability of the salt cavern, the volume of the plastic zone is statistically analysed.

Table 5 lists the variation of the plastic zone in different salt cavern schemes. Data in Table 5 indicate that the plastic zone is enlarged first and then reduces with time, and the plastic zone obtained in each scheme reaches the maximum value after five years of operation. This is mainly attributed to the stress redistribution caused by the water solution and initial operation of the salt cavern. The plastic deformation expands gradually with time. When the shear stress exceeds the strength of the salt rock, the plastic zone develops. The creep of the salt rock tends to a steady-state as the operation continues, and the creep rate is less than that of the initial creep stage, this will leads to the reduction of the plastic zone.

**Table 5:** The change of plastic zone under different schemes of gas storage year by year injection and production (10^3^ m^3^).

Simulation number	Time	Injection	Production	Injection	Production	Injection	Production
		a (benchmark scheme)		b		c	
× 1 (IGP range)	1	4.2436	4.2437	3.6233	3.6240	5.6752	5.6753
	5	4.2453	4.2454	3.6252	3.6253	5.6767	5.6770
	10	4.2447	4.2447	3.6246	3.6246	5.6766	5.6764
	20	4.2420	4.2418	3.6215	3.6214	5.6741	5.6738
	30	4.2375	4.2372	3.6161	3.6159	5.6699	5.6695
× 2 (minimum operation IGP)	1	4.2436	4.2437	3.6231	3.6240	5.6752	5.6754
	5	4.2453	4.2454	3.6252	3.6253	5.6767	5.6770
	10	4.2447	4.2447	3.6247	3.6247	5.6764	5.6764
	20	4.2420	4.2418	3.6220	3.6219	5.6735	5.6733
	30	4.2375	4.2372	3.6172	3.6170	5.6688	5.6684
× 3 (Dwell time of the minimum IGP)	1	4.2436	4.2437	4.2436	4.2448	4.2436	4.2437
	5	4.2453	4.2454	4.2460	4.2459	4.2460	4.2459
	10	4.2447	4.2447	4.2456	4.2455	4.2455	4.2454
	20	4.2420	4.2418	4.2432	4.2430	4.2430	4.2428
	30	4.2375	4.2372	4.2392	4.2389	4.2388	4.2384
× 4 (cycle IGP)	1	4.2436	4.2437	4.2720	4.2718	4.3165	4.3163
	5	4.2453	4.2454	4.2736	4.2734	4.3181	4.3179
	10	4.2447	4.2447	4.2730	4.2727	4.3175	4.3173
	20	4.2420	4.2418	4.2699	4.2696	4.3147	4.3144
	30	4.2375	4.2372	4.2648	4.2644	4.3099	4.3094

**Figure 13:**
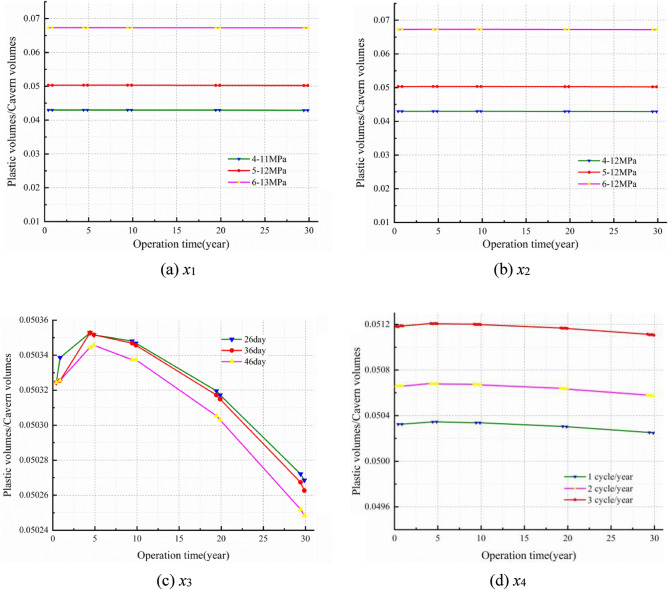
The ratio of the plastic zone volume to the salt cavern volume in different schemes.

Figure 13 presents the ratio of the plastic zone volume to the salt cavern volume of different schemes for 30 years. Based on Fig. 13, we can see that the curves of the ratio with time are approximately linear in schemes X1 and X2, which suggest that the duration of time barely affect the plastic zone volume, while the change of the injection-production operation pressure has a relatively great effect on the volume. However, the curves have a convex shape in schemes X3 and X4, which signify that the injection-production time and the pressure all has a remarkable effect on the volume of the plastic zone. And the plastic zone is most significantly influenced by the injection-production time in scheme X3.

After 30 years of operation, in scheme X1, the ratio of the plastic zone to the salt cavern obtained with the IGP ranges are 5–12 MPa and 6–13 MPa is increased by 17.18% and 56.80%, respectively, compared with that obtained when the IGP range is 4–11 MPa. In scheme X2, the ratio obtained with the IGP ranges are 5–12 MPa and 6–12 MPa is increased by 17.15% and 56.71%, respectively, compared with that obtained when the IGP range is 4–12 MPa. In scheme X3, the ratio obtained with the minimum IGP residence time are 36 days and 46 days is reduced by 0.01% and 0.04%, respectively, compared with that obtained when the residence time is 26 days. In scheme X4, the ratio obtained with the injection-production cycle are twice and three times one year is increased by 0.64% and 1.71%, respectively, compared with that obtained when the cycle is once a year. This results show that the volume of the plastic zone is enlarged with the increase of the IGP interval, minimum IGP, and injection-production cycle, while it is reduced with the extension of the minimum IGP residence time. And the variation becomes remarkably with the increase of parameters.

## Sensitivity analysis of injection-production operation parameters

**Figure 14:**
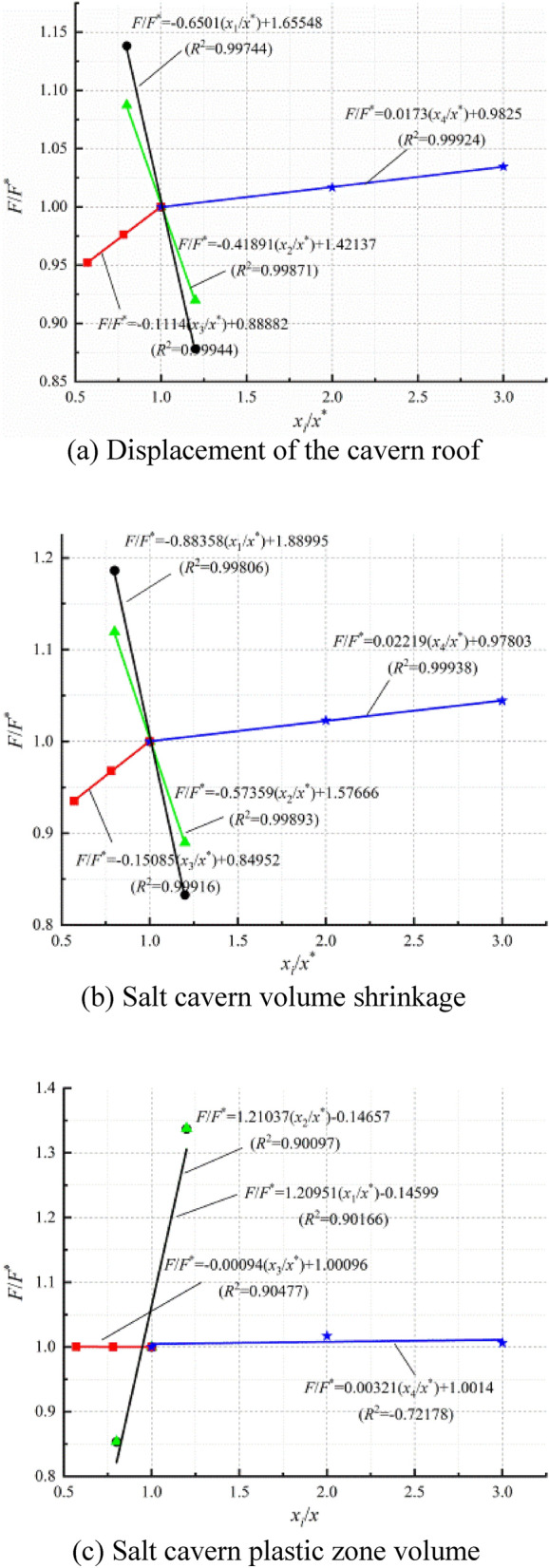
Comparison diagram of sensitivity coefficient of injection-production operation parameters.

The sensitivity analysis method is employed to study the influence of the injection-production operation parameters on the stability of the salt cavern in operation. Sensitivity analysis methods are divided into the single-factor and the multifactor sensitivity analysis^50,51^. In this study, the single-factor sensitivity analysis method is employed for the dimensionless treatment of the operation parameters. The curves of F/F* and x_i_/x* (i = 1, 2, 3, …, n) are plotted in Fig. 14. The abscissa x_i_/x* represents the ratio of the injection-production parameters of each model to the benchmark scheme, and the ordinate F/F* represents the ratio of the system characteristics (displacement at the roof of the cavern, volume shrinkage and plastic zone volume) of each model to that of the benchmark scheme, and the absolute value of the curve slope is defined as the sensitivity coefficient. The sensitivity coefficient reflects the impact of each injection-production parameter on the stability of the salt cavern during the injection-production process. The higher the sensitivity coefficient is, the greater the influence of this parameter on the stability of the salt cavern.

**Figure 15:**
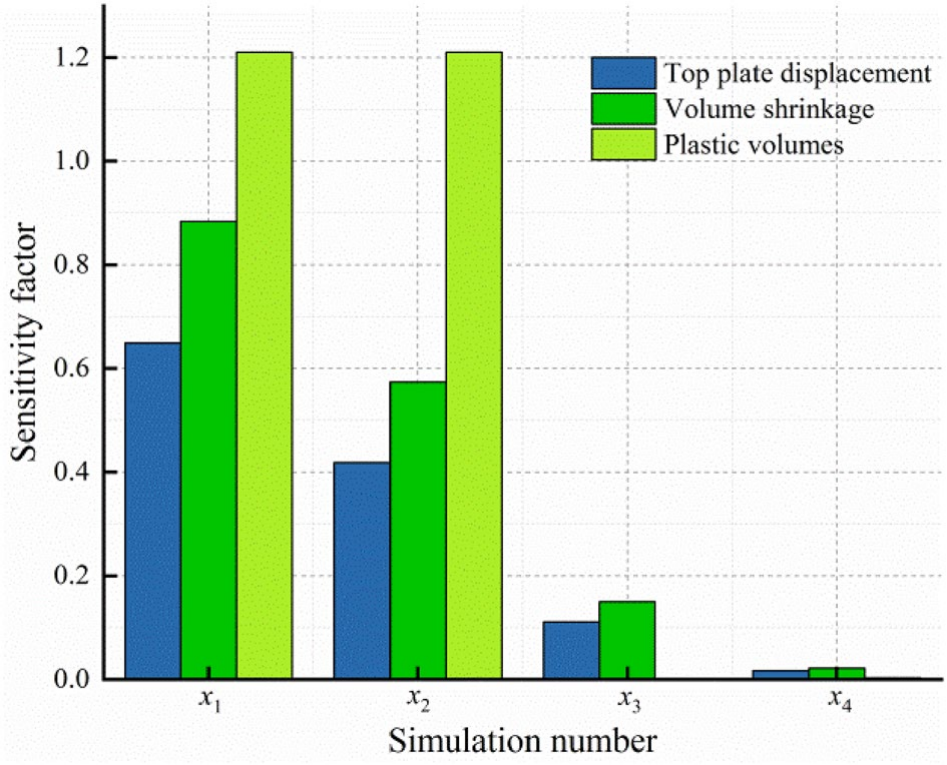
Sensitivity coefficient comparison.

Figure 15 shows a comparison of the sensitivity coefficients of the injection-production operation parameters. It can be seen from Figs. 14 and 15 that when only the influence of one certain system characteristic is considered, the displacement at the cavern roof, the volume shrinkage, and the plastic zone of the salt cavern are significantly affected by the IGP interval (*x*_1_) , and the sensitivity coefficient is 0.6501, 0.88358, and 1.21037, respectively. The displacement is affected a lesser extent by the minimum IGP (*x*_2_). The residence time of the minimum IGP (*x*_3_) and cycle IGP (*x*_4_) has little effect on the plastic zone of the salt cavern. The sensitivity coefficients of each injection-production parameter, from large to small, are ranked as follow: IGP interval (*x*_1_) > minimum IGP (*x*_2_) > residence time of the minimum IGP (*x*_3_) > cycle IGP (*x*_4_). Based on the actual operation of the salt cavern, the number of the injection-production cycle is always limited to 1–3 times one year. Therefore, the effect of the number of injection-production cycles on the stability of the salt cavern is limited. Compared with other injection-production parameters, the injection-production cycle (*x*_4_) has fewer influence on the stability of the salt cavern during operation.

## Conclusions

To achieve the rational allocation of the injection-production operation parameters, the influence of the minimum IGP, IGP interval, minimum IGP residence time, and injection-production cycle on the safety and stability of the layered salt cavern in the operation period is investigated. According to the comparison of numerical simulation schemes, the following conclusions are proposed:

1. The deformation of the surrounding rock in the near field of the cavern wall changes significantly with the development of the injection-production. The displacement at each point of the cavern wall is different, and the maximum value appears at the cavern roof. The displacement of the surrounding rock is reduced significantly with the enhance of the IGP interval and minimum IGP, while it is augmented with increasing the minimum IGP residence time and injection-production cycle, and the variation tendency becomes more obvious over time.
2. The expansion coefficient of the surrounding rock becomes greater with the raise of the IGP range and the minimum IGP, while it decreases with increasing the minimum IGP residence time and the injection-production cycle. Conversely, the volume shrinkage rate of the salt cavern is reduced with enhancing the IGP interval and minimum IGP, while it is increased with the increment of the minimum IGP residence time and the injection-production cycle.The volume shrinkage rates of salt cavern in different cases increase with time, and the trend slowed down gradually.
3. The plastic zone is enlarged first and then reduced with time, and the plastic zone obtained in each scheme reached the maximum after five years of operation. The volume of the plastic zone is enlarged with the increment of the IGP interval, minimum IGP, and injection-production cycle, while it is reduced with the extension of the minimum IGP residence time. And the variation becomes remarkably with the increase of parameters.
4. The sensitivity of the long-term operation of the salt cavern to various injection-production operation parameters is different. The order of sensitivity coefficient of each parameter is, in decreasing order, are sorted as follow: IGP interval, minimum IGP, minimum IGP residence time, injection-production cycle.
5. For optimization of the injection-production operation of the salt cavern, it is suggested that the minimum pressure or the operation pressure interval should be enhanced, and the number of the injection-production cycle should be increased at the same time. And then realize the improvement of the quality and efficiency of the injection-production operation.

### Supplementary Information

Supplementary Information 1.

## Data Availability

The data used to support the findings of this study are available from the corresponding author upon request.
