# Supplementary material for: Sensitivity analysis of operation parameters of the salt cavern under long-term gas injection-production
Source: Sci Rep. 2023 Nov 16;13:20012. doi: 10.1038/s41598-023-47352-w (PMC10654732; doi:10.1038/s41598-023-47352-w)
Supplement: Supplementary file 1 — Supplementary Information 1. [file 41598_2023_47352_MOESM1_ESM.zip › Supplementary file/Numerical Simulation Algorithm with Sequential Steps.docx]

Numerical Simulation Algorithm with Sequential Steps

1. Numerical Model

Through WinUbro software, the cavity is simulated and transformed into Flac3D. file ‘Flacmodel_haitang.Flac3D’, and then the three-dimensional finite element geomechanical model is established by FLAC3D software.

1. Numerical Simulation

The numerical simulation of the gas storage model is carried out by using the CALL command in Flca3D software to run the static cavity command flow e.g. ‘ny_4_zc’. After the calculation of file ‘ny_4_zc.sav’, the numerical simulation of injection and production operation for 30 years is carried out. Each scheme is operated according to this.

1. Results Cloud and Data Extraction

The software Tecplot3.0 was used to post-process the numerical simulation results, and the deformation contours and Safety factor isolines of salt cavern were derived respectively. The displacement values of the top of the gas storage chamber, the volume of the cavity and the area of the plastic zone of the surrounding rock with different operating years were extracted.

1. Analysis of Effect

The displacement of the cavern roof, salt cavern volume shrinkage and salt cavern plastic zone volume were obtained by processing the data, and the sensitivity analysis was carried out.
